# Learning defects in *Drosophila* growth restricted *chico* mutants are caused by attenuated adenylyl cyclase activity

**DOI:** 10.1186/s13041-016-0217-3

**Published:** 2016-04-06

**Authors:** Shintaro Naganos, Kohei Ueno, Junjiro Horiuchi, Minoru Saitoe

**Affiliations:** Tokyo Metropolitan Institute of Medical Science, 2-1-6, Kamikitazawa, Setagaya, 185-8506 Tokyo Japan

**Keywords:** Insulin signaling, cAMP, Synaptic plasticity, Associative learning, Intrauterine growth retardation

## Abstract

**Background:**

Reduced insulin/insulin-like growth factor signaling (IIS) is a major cause of symmetrical intrauterine growth retardation (IUGR), an impairment in cell proliferation during prenatal development that results in global growth defects and mental retardation. In *Drosophila*, *chico* encodes the only insulin receptor substrate. Similar to other animal models of IUGR, *chico* mutants have defects in global growth and associative learning. However, the physiological and molecular bases of learning defects caused by *chico* mutations, and by symmetrical IUGR, are not clear.

**Results:**

In this study, we found that *chico* mutations impair memory-associated synaptic plasticity in the mushroom bodies (MBs), neural centers for olfactory learning. Mutations in *chico* reduce expression of the *rutabaga*-type adenylyl cyclase (*rut*), leading to decreased cAMP synthesis in the MBs. Expressing a *rut*^+^ transgene in the MBs restores memory-associated plasticity and olfactory associative learning in *chico* mutants, without affecting growth. Thus *chico* mutations disrupt olfactory learning, at least in part, by reducing cAMP signaling in the MBs.

**Conclusions:**

Our results suggest that some cognitive defects associated with reduced IIS may occur, independently of developmental defects, from acute reductions in cAMP signaling.

## Background

Intrauterine growth retardation (IUGR) causes growth and psychological defects in children, and can be categorized into symmetrical and asymmetrical types [1]. Symmetrical IUGR is caused by early intrauterine disruptions, which lead to global decreases in cell proliferation, reduced brain size and cognitive deficits [2]. Asymmetrical IUGR is caused by poor nutrition or oxygen supply to the fetus during the third trimester of pregnancy, leading to decreased body size, but normal or near normal brain sizes. Symmetrical IUGR phenotypes include reduced numbers of neurons in the hippocampus and the cerebellum in neonates [3], and these reductions are proposed to explain cognitive deficits observed in symmetric IUGR. However, the actual mechanisms causing cognitive defects upon symmetrical IUGR are still unknown.

Mutations in insulin/insulin-like growth factor signaling (IIS) are associated with symmetrical IUGR [4, 5]. In *Drosophila*, homozygous mutations in the *chico* gene, which encodes the only insulin receptor substrate in flies, decrease cell proliferation, reduce global body size and cause frequent death at birth [6–8]. Although *chico* mutants also display impaired associative learning [9], the physiological and molecular bases of learning defects caused by *chico* mutations are not clear.

During *Drosophila* olfactory aversive learning, flies learn to associate an odor with aversive electric shocks [10]. Olfactory associations are formed in central brain areas called the mushroom bodies (MBs) [11]. Information about the odor or conditioned stimulus (CS), is transmitted to the MBs by cholinergic projection neurons (PNs) from the antennal lobes (ALs) [12–14], while information about the electric shocks or unconditioned stimuli (US), are proposed to be transmitted to the MBs by dopaminergic neurons, which receive somatosensory information from the ascending fibers of the ventral nerve cord (AFV) [15–19]. Previously, using functional imaging of dissected, cultured brains, we showed that simultaneous stimulation of AL and AFV inputs to the MBs induces long-term enhancement (LTE) of synaptic transmission from the ALs to the MBs. We further showed that this LTE is a likely cellular correlate of olfactory aversive memory [20].

In this study, we found that *chico* mutants are defective for LTE, and have decreased expression of the *rutabaga* (*rut*) gene. *rut* encodes an adenylyl cyclase (*rut*-AC) required for cAMP signaling during associative learning. *rut* mutants have impaired learning [10, 21], and we show that expression of a *rut*^*+*^ transgene in the MBs restores both learning and LTE in *chico* mutants. *rut*^*+*^ expression restores learning and LTE without increasing numbers of MB neurons, indicating that learning defects in *chico* mutants are caused by reduced cAMP signaling, and not by reduced cell number. This suggests that some of the cognitive defects caused by symmetrical IUGR may occur independently of reduced cell number.

## Results

### LTE is suppressed in *chico*^*1*^ mutants

**Fig. 1:**
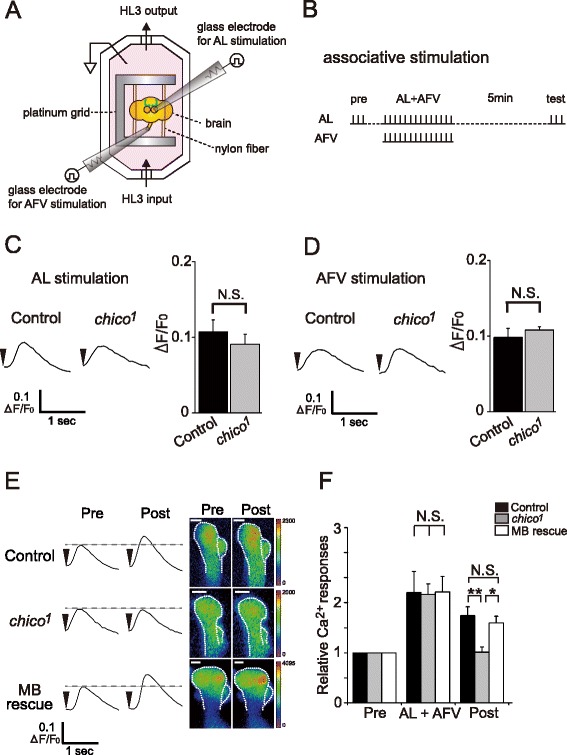
*Chico* expression in the MBs is required for LTE of AL induced MB responses. **a** Schematic diagram of the recording setup. A dissected brain, held on a platinum grid by nylon fibers, is placed in a recording chamber perfused with HL3. Glass electrodes are used to electrically stimulate the AL and AFV, and resulting fluorescent signals are measured using a confocal microscope. See Materials and Methods for details. **b** Stimulation protocol. AL-induced Ca^2+^ responses before (pre) and after (test/post) simultaneous AL and AFV stimulation (AL + AFV) were used to quantify LTE. Vertical tick marks represent AL or AFV stimulation. **c** and **d** Typical traces of AL-induced (**c**) and AFV-induced (**d**) Ca^2+^ responses in control (*UAS-G-CaMP/+; OK107/+*) and *chico* (*chico* ^*1*^ *; UAS-G-CaMP/+; OK107/+*) brains, and summary graphs. *N* = 6 for all data. **e** Typical AL-induced Ca^2+^ responses at the distal end of α/α’ lobes of the MBs before (pre) and after (post) simultaneous AL and AFV stimulation. Scale bar, 10 μm. **f** Summary of Ca^2+^ responses obtained from control, *chico* ^*1*^ and MB-*chico* rescue (*chico* ^*1*^ *,UAS-chico/chico* ^*1*^ *; UAS-G-CaMP/+; OK107/+*) brains. Arrowheads indicate stimulation onset. *N* = 6 for each data point. ** *P* < 0.01, * *P* < 0.05, N.S. Not Significant. Data are shown as means ± SEM

*Chico* mutants are defective for olfactory learning [9]. To determine whether they are also defective for memory-associated plasticity, we examined LTE in brains of *chico* mutants expressing the Ca^2+^ indicator G-CaMP 1.3 in the MBs (Fig. 1a). *chico*^*1*^ mutations consist of a P-element insertion 80 bp downstream from the translational start site, resulting in a complete loss of protein [6, 9]. LTE is typically observed at the distal ends of the vertical lobes (α/α’) of the MBs, and is induced upon simultaneous stimulation of the ALs and AFV (AL + AFV in Fig. 1b), but not upon AL or AFV stimulation alone [20].

Ca^2+^ responses in the MBs induced by AL or AFV stimulation alone were indistinguishable between *chico*^*1*^ and control brains (Fig. 1c and d). However, the increase in AL-induced Ca^2+^ responses after simultaneous stimulation of the AL and AFV (AL + AFV), observed in the control brains (*UAS-G-CaMP/+; OK107/+*), was suppressed in *chico* brains (*chico*^*1*^*; UAS-G-CaMP/+; OK107/+*) (Fig. 1e and f). This indicates that basal synaptic transmission from the ALs and AFV to the MBs is unaffected in *chico* mutants, but LTE is disrupted. Olfactory learning is restored in *chico*^*1*^ flies by expressing a *chico*^*+*^ transgene in the MBs [9]. Consistent with this, expressing a *chico*^*+*^ transgene in the MBs (*chico*^*1*^*,UAS-chico; UAS-G-CaMP/+; OK107/+*) also restored LTE in *chico*^*1*^ brains (Fig. 1e and f), demonstrating that defects in LTE are caused by reduced *chico* expression in the MBs.

### *Chico* mutations decrease expression of *rutabaga* and synthesis of cAMP in the MBs

**Fig. 2:**
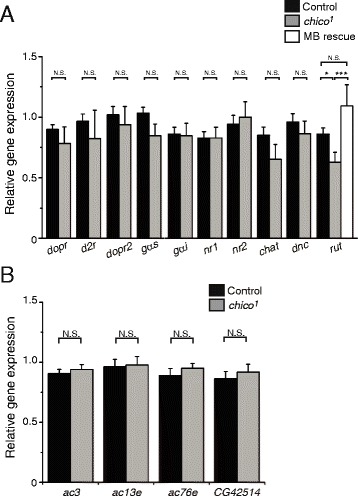
*Rutabaga* expression is reduced in *chico* mutants. **a** Quantitated mRNA amounts in control (*black*), *chico* ^*1*^ homozygote (*gray*) and MB-*chico* rescued (*chico* ^*1*^ *, UAS-chico* ^*+*^ */chico* ^*1*^ *; OK107/+*) fly head extracts. *N* > 5 for each data point. **b** Expression of other putative adenylyl cyclases, encoded by *ac3*, *ac13e*, *ac76e* and *CG42514*, is not altered in *chico* ^*1*^ mutants. *N* = 8. All data in this figure are normalized to total RNA, and are shown as means ± SEM. *** *P* < 0.001, * *P* < 0.05, N.S. Not Significant

We previously demonstrated that AL-induced Ca^2+^ responses in the MBs are mediated by nicotinic acetylcholine receptors (nAChRs), while AFV-induced Ca^2+^ responses in the MBs are mediated by NMDA receptors (NRs). Besides nAChRs and NRs, a third type of receptor, the D1-type dopamine receptor (D1R), encoded by the *dopr* gene, is also required for LTE formation [20]. To investigate the molecular bases of LTE defects in *chico* mutants, we examined expression of genes associated with these receptors (Fig. 2a). As expected from the normal AL- and AFV-induced Ca^2+^ responses in *chico* brains (Fig. 1c and d), expression of NRs (*nr1* and *nr2*), and choline acetyltransferase (*chat*), which is required for synthesis of acetylcholine, was unaltered in *chico* mutants. Likewise, expression of *dopr*, a second D1R subtype encoded by *dopamine receptor 2* (*dopr2*), and the D2-type dopamine receptor encoded by *d2-like dopamine receptor* (*d2r*), were also unchanged in *chico* mutants.

Cholinergic, glutamatergic and dopaminergic inputs affect various downstream signaling pathways, including the cAMP pathway. In particular, an adenylyl cyclase (AC) encoded by the *rutabaga* (*rut*) gene and a phosphodiesterase (PDE) encoded by the *dunce* (*dnc*) gene are preferentially expressed in the MBs, and play critical roles in olfactory learning and synaptic plasticity [10, 22, 23]. While we did not observe significant changes in expression of *dnc*, or of the G-protein Gs alpha subunit (*gαs*) or the Gi alpha subunit (*gαi*), we found a significant decrease in *rut* expression in *chico* mutants compared to controls. Furthermore, expression of a *chico*^*+*^ transgene in the MBs restored *rut* expression to control levels in *chico* flies. *Drosophila* have 12 other adenylyl cyclase genes besides *rut* [24], four of which show high expression in the brain [25]. However, we did not observe significant changes in expression of these other brain-expressed AC genes in *chico* mutants compared to wild-type (Fig. 2b), indicating that *chico* specifically regulates expression of *rut*-AC.

**Fig. 3:**
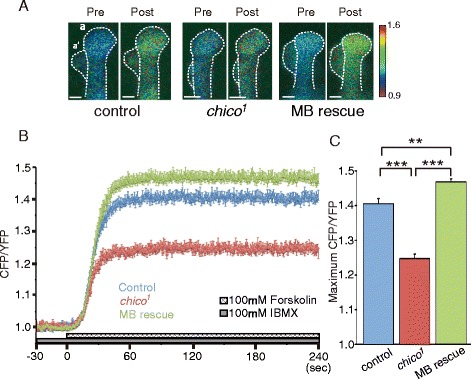
Adenylyl cyclase-dependent cAMP synthesis is reduced in *chico* ^*1*^ mutants. **a** Typical FRET images observed at the distal end of the α/α’ MB lobe 10 sec before (pre), and 60 sec after (post), forskolin stimulation in brains from control (*UAS-Epac1-camps/+; OK107/+*), *chico* ^*1*^ (*chico* ^*1*^ *; UAS-Epac1-camps/+; OK107/+*) and MB-*chico* rescue (*chico* ^*1*^ *,UAS-chico/chico* ^*1*^ *; UAS-Epac1-camps/+; OK107/+*) flies. A shift towards red indicates an increased CFP/YFP ratio and increased cAMP amounts. Scale bar, 10 μm. **b** Time course of FRET changes (CFP/YFP) during forskolin stimulation. **c** Summary of maximum FRET changes (maximum CFP/YFP) upon forskolin stimulation. *N* = 14 for control lines, *N* = 13 for *chico* ^*1*^ mutants and MB rescue lines. *** *P* < 0.001, and ** *P* < 0.01. Data are shown as means ± SEM

Decreases in *rut* expression suggest that *chico* mutants may be defective for cAMP synthesis in the MBs. To test this possibility, we expressed a fluorescence resonance energy transfer (FRET) based cAMP probe, Epac1-camps [26, 27], in the MBs (Fig. 3). Using this probe, intracellular cAMP levels can be monitored as increases in the CFP/YFP ratio (decreasing FRET signal). When we applied 100 μM forskolin, an activator of AC, in the presence of IBMX, a cyclic nucleotide phosphodiesterase inhibitor, we observed a robust change in FRET signals in the vertical lobes (α/α’ lobes) of the MBs of control brains (*UAS-Epac1-camps/+; OK107/+*) (Fig. 3a and b). In *chico* mutant brains (*chico*^*1*^*; UAS-Epac1-camps/+; OK107/+*), this change was significantly attenuated after the same treatment (Fig. 3a and b). Furthermore, this attenuated signal was rescued by expressing a *chico*^*+*^ transgene in the MBs (*chico*^*1*^*,UAS-chico; UAS-Epac1-camps/+; OK107/+*) (Fig. 3a–c). These results suggest that *chico* regulates cAMP production in the MBs.

### Expression of a *rut*^*+*^ transgene in the MBs rescues learning defects of *chico* mutants

**Fig. 4:**
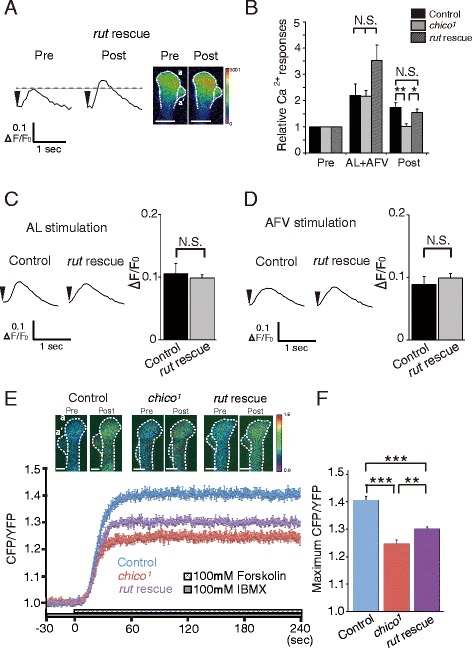
MB expression of a *rut* ^*+*^ transgene is sufficient to restore LTE formation and increase cAMP synthesis in *chico* mutants. **a**, **b** MB expression of the *rut* ^*+*^ transgene restores LTE in *chico* mutants. **a** Typical AL-induced Ca^2+^ responses before (pre) and 5 min after (post) AL + AFV stimulation in *chico* ^*1*^ *, UAS-rut/chico* ^*1*^ *; UAS-G-CaMP/+; OK107/+* (*rut* rescue) brains. Scale bar, 5 μm. **b** Summary of Ca^2+^ responses in control, *chico* ^*1*^ and *rut* rescued flies. **c**, **d** Typical traces (*left*) and average maximal responses (*right*) of AL-induced (**c**) and AFV-induced (**d**) Ca^2+^ responses in the MBs of *chico* ^*1*^ (control) and *rut* rescue brains. *N* = 6 for all data in **a**–**d**. **e, f** MB expression of a *rut* ^*+*^ transgene increases cAMP synthesis in *chico* ^*1*^ mutants upon forskolin stimulation. **e** Typical FRET images and time course (normalized CFP/YFP) upon forskolin stimulation in control (*UAS-Epac1-camps/+; OK107/+*, *N* = 7), *chico* ^*1*^ (*chico* ^*1*^ *; UAS-Epac1-camps/+; OK107/+*, *N* = 8) and *rut* rescue (*chico* ^*1*^ *, UAS-rut/chico* ^*1*^ *; UAS-Epac1-camps/+; OK107/+*, *N* = 6) brains. Scale bar, 10 μm. **f** Summary of maximum CFP/YFP ratios upon forskolin stimulation. N.S. Not Significant, ***P < 0.001, **P < 0.01. Data are shown as means ± SEM

**Fig. 5:**
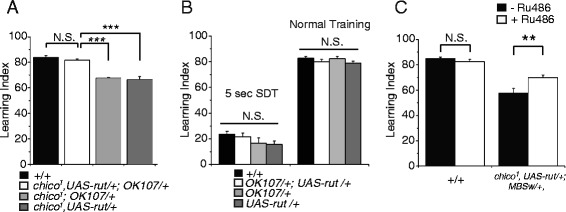
MB expression of a *rut* ^*+*^ transgene ameliorates learning defects of *chico* mutants. **a** MB expression of a *rut* ^*+*^ transgene rescues learning (3 min memory) in *chico* mutants. *N* = 8. **b** Learning (3 min memory) is unaffected by MB overexpression of a *rut* ^*+*^ transgene in a wild type background. No significant differences in performance were observed after either 5 sec short duration training (SDT) or 60 sec normal training. *N* = 7–14. **c** Learning is improved in *chico* mutants by expressing a *rut* ^+^ transgene from a MB-Geneswitch driver. *N* = 8. N.S. Not Significant, *** *P* < 0.001, **P < 0.01. All Data in this figure are shown as means ± SEM

We next tested whether decreased *rut* expression in the MBs could be responsible for impaired LTE, cAMP production and learning in *chico* mutants. When a *rut*^*+*^ transgene was expressed in the MBs, LTE in *chico* mutants was restored to wild-type levels (Fig. 4a and b), while Ca^2+^ responses evoked by AL- or AFV stimulation alone were not affected (Fig. 4c and d). Forskolin-induced Epac1-camps FRET signals in *chico* mutants were also partially restored by MB expression of the *rut*^*+*^ transgene (Fig. 4e and f). Furthermore, MB expression of the *rut*^*+*^ transgene rescued learning (measured 3 min after conditioning) in *chico* flies (Fig. 5a). Taken together, these results indicate that decreased *rut* expression in the MBs is the likely cause of the reduced cAMP production, and impaired LTE and learning observed in *chico* mutants.

A previous study has reported that overexpressing AC can enhance memory retention in wild-type mice [28], raising an alternate possibility that MB expression of the *rut*^*+*^ transgene may enhance olfactory learning in general, through a mechanism unrelated to *chico* rescue. Although we did not observe an increase in learning upon MB overexpression of the *rut*^*+*^ transgene in a wild-type background (Fig. 5b, right panel), normal 1 min-duration olfactory conditioning induces a maximal, plateau-level amount of learning in wild-type flies, where increases may not be observable [9, 29, 30]. Thus, we employed short, 5 sec-duration conditioning trials, which produce lower learning indices, to assess learning enhancement upon *rut*^*+*^ overexpression in an otherwise wild-type background. As seen in the left panel of Fig. 5b, learning after 5 sec conditioning does not increase in flies overexpressing *rut*^*+*^ in the MBs (*UAS-rut/+;OK107/+*). These results indicate that MB expression of *rut*^*+*^ does not enhance learning in general, but specifically rescues learning defects in *chico* flies.

To address whether decreased *rut* expression in the MBs specifically at the adult stage causes learning defects in *chico* flies, we acutely expressed the *rut*^+^ transgene from a MB-Geneswitch driver [31]. When *chico* flies were fed RU for 4 days prior to training, we observed partial but significant restoration of learning (Fig. 5c), suggesting that decreased *rut* activity in adult *chico* MBs contributes to learning defects in *chico* flies.

### MB expression of *rut*^*+*^ does not restore MB growth in *chico* mutants

**Fig. 6:**
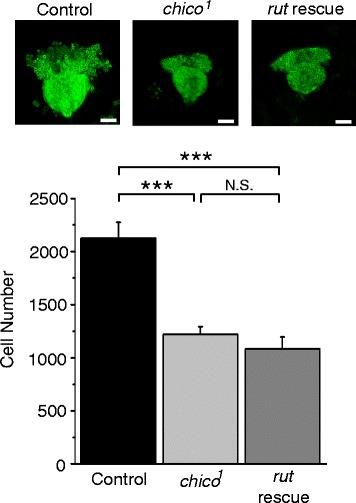
MB expression of a *rut* ^*+*^ transgene does not restore Kenyon cells numbers in *chico* homozygotes. Top panel, typical fluorescent images of the calyx and Kenyon cells in control (*UAS-mCD8::GFP/+; OK107/+)*, *chico* ^*1*^ (*chico* ^*1*^ *, UAS-rut* ^*+*^ */chico* ^*1*^ *; UAS-mCD8::GFP/+; OK107/+*) and *rut* rescue (*chico* ^*1*^ *, UAS-rut* ^*+*^ */chico* ^*1*^ *; UAS-mCD8::GFP/+; OK107/+*) brains. Scale bars represent 20 μm. Bottom panel, quantification of the number of kenyon cells per hemisphere in indicated lines. *N* = 6. *** *P* < 0.001, N.S. Not Significant. Data are shown as means ± SEM

Both learning defects and reduced Kenyon cell (MB intrinsic cell) number in *chico* mutants are rescued by expressing a *chico*^*+*^ transgene in the MBs [9]. To determine whether the rescue of *chico* LTE and learning defects by MB *rut*^*+*^ expression is also associated with increases in Kenyon cell number, we counted numbers of Kenyon cells in wild-type, *chico*^*1*^ and *chico*^*1*^ MB *rut*^*+*^ rescue brains. As seen in Fig. 6, *chico*^*1*^ mutants have reduced numbers of Kenyon cells and MB *rut*^*+*^ expression does not rescue this phenotype. Thus, cAMP signaling and learning defects in *chico* mutants occur independently of defects in cell proliferation, and at least some of the learning defects observed in *chico* mutants can be rescued in the absence of increasing cell number.

## Discussion

*Drosophila chico* mutations and symmetrical IUGR are associated with several similar phenotypes, including decreased cell proliferation, global growth restriction, significant lethality at birth and learning defects [6, 9]. In this study, we investigated the physiological and molecular bases of learning defects caused by *chico* mutations, and found that mutants are defective for synaptic plasticity, measured as LTE of AL-induced MB responses. *chico* mutants have normal olfactory avoidance and shock reactivity [9], and normal basal AL-induced and AFV-induced Ca^2+^ responses in the MBs, suggesting that they are defective for forming associations rather than transmitting sensory information. Expression of a *chico*^*+*^ transgene in the MBs restores both olfactory learning and LTE in *chico* mutants. Thus, we propose that impaired LTE is likely to be physiologically correlated with learning defects in *chico* mutants.

We further found that *chico* mutants express significantly reduced amounts of *rut*. In comparison, expression of various other genes, including those involved in dopaminergic, glutamatergic and cholinergic signaling, is normal. Expressing a *chico*^*+*^ transgene in the MBs restores normal *rut* expression, and normal cAMP levels in *chico* MBs, suggesting that *chico* likely regulates *rut* in a cell autonomous manner.

Rut-AC and its mammalian orthologue, type I AC, play critical roles in memory-associated plasticity and memory retention [10, 32, 33]. In flies, *rut* expression, specifically in the MBs, is required for associative learning [33, 34]. Consistent with this, we found that restoring Rut-AC function in the MBs is sufficient to partially restore forskolin-induced cAMP synthesis, and completely restore LTE and olfactory learning in *chico* mutants.

MB *rut* expression, specifically in adults, is required for adult olfactory learning [31, 35], and we also found that acute *rut*^+^ expression in adults significantly improves learning in *chico* mutants. Previously, it has been reported that increasing cAMP amounts increases synaptic transmission at the *Drosophila* larval neuromuscular junction [23]. However, expressing *rut*^*+*^ in the MBs did not affect basal AL-induced and AFV-induced Ca^2+^ responses in the MBs, suggesting that Rut-AC, which is activated by both G-protein and Ca^2+^/calmodulin signaling [32], functions during detection of coincident sensory stimuli, rather than during simple synaptic transmission.

Although increasing type I AC activity enhances memory in mice [28], we found that expressing a *rut*^*+*^ transgene in the MBs does not enhance 3-min memory in flies from a wild-type background. Thus, AC seems to be a limiting factor in learning and memory formation in mice, while MB *rut*-AC amounts do not seem to be limiting in flies. Consistent with this idea, we found that expressing a *rut*^*+*^ transgene in the MBs at levels that only partially restore cAMP amounts, is enough to fully rescue LTE and learning defects in *chico* mutants. We propose that the 27 % reduction in *rut*-AC expression observed in *chico* mutants (Fig. 2) reduces AC protein amounts below a threshold, such that insufficient amounts of cAMP are produced after coincident stimulation. This leads to deficiencies in both LTE and learning. Mild transgenic expression of *rut*^*+*^ in the MBs may not restore *rut* expression to wild-type levels, but does increase expression enough to restore normal LTE and learning.

It is unclear why transgenic expression of *rut*^*+*^ was insufficient to completely restore cAMP amounts to wild-type levels in *chico* mutants, since we expressed *rut*^*+*^ from a strong *OK107* driver. Since the *chico* mutation affects endogenous *rut* expression, it is possible that *chico* also affects expression of other genes including expression from the *OK107* promoter. In addition, insulin signaling affects the target of rapamycin (TOR) kinase, which regulates protein translation [36, 37]. Thus *chico* may affect both transcription and translation, such that *rut*^*+*^ transgene expression only partially rescues *chico* defects.

Although cAMP signaling is known to regulate IIS [38, 39], there have only been a few previous studies of IIS affecting cAMP. Of these, all have focused on IIS regulation of cAMP degradation by phosphodiesterases [40, 41]. Ours is the first to report that IIS regulates cAMP production by controlling adenylyl cyclase expression. IIS activates the AKT kinase, which then phosphorylates and inactivates the forkhead transcription factor, Foxo [36, 42]. Thus it is possible that reduced IIS activates Foxo, which in turn induces expression of a transcriptional repressor of *rut*. It will be of great interest in the future to determine whether IIS regulates *rut* expression through Foxo dependent or independent mechanisms.

## Conclusions

We found that *chico* mutations impair learning and synaptic plasticity due to reduced expression and activity of the Rut-AC in the MBs. Attenuated AC activity is also associated with human IUGR, and stimulation-dependent cAMP production is defective in cultured fibroblasts derived from human patients with pre- and postnatal growth retardation, mental retardation and insulin resistance [43]. Given these similarities, our results may provide novel insights into the causes of mental retardation associated with symmetrical IUGR.

## Methods

### Fly stocks

All fly stocks were maintained at 25 ± 2 °C and 60 ± 10 % humidity under a 12 h/12 h light dark cycle. All transgenic flies and mutants were outcrossed to our wild-type control line *w(CS10)* [44] between 5 and 10 times. For imaging analyses, we used female flies, since *chico* homozygous flies are semi-lethal and homozygous males rarely emerged in our genetic background. For behavioral tests, we used both male and female flies.

### Imaging analysis

#### Imaging preparation

Brains with attached ventral nerve cords (VNC) were dissected in 0 mM Ca^2+^ HL3 medium [NaCl, 70 mM; sucrose, 115 mM; KCl, 5 mM; MgCl_2_, 20 mM; NaHCO_3_, 10 mM; trehalose, 5 mM; and HEPES, 5 mM, pH 7.3] [45]. The ventral nerve cord was then cut at the cervical connective and isolated brains were immobilized by placing their optic lobes between two nylon fiber bundles stretched across a platinum grid, and placed in a bath chamber (Fig. 1a). ALs and AFV were electrically stimulated using glass micro-electrodes and stimulators (SEN-7103, Nihon Kohden, Japan) and isolators (SS-104 J for AL, SS202J for AFV, Nihon Kohden). During experiments, fresh HL3 [NaCl, 70 mM; sucrose, 115 mM; KCl, 5 mM; MgCl_2_, 20 mM; CaCl_2_, 1.5 mM; NaHCO_3_, 10 mM; trehalose, 5 mM; and HEPES, 5 mM, pH 7.3] was infused into the chamber using a peristaltic pump (3 ml/min Gilson, Inc.).

#### Ca^2+^ imaging

Images were captured using a confocal microscope system (A1, Nikon Corp., Japan). The objective lens was a 20 × water-immersion lens (NA: 0.5 Nikon Corp.). Before experiments, we set the offset value so that the background intensity would be close to 0. The *F* value was calculated for each pixel in the region of interest (ROI) using NIS-elements software (NIS-Elements Ar, Nikon Corp.).

We obtained initial fluorescence values (F_0_) by averaging values in the five sequential frames before stimulation onset. To record AL- and AFV-induced Ca^2+^ responses, we stimulated the AL or AFV with 3 trains of 30 pulses (100 Hz, 1.0 ms pulse duration, at a current that produced a 10 % increase in fluorescence compared to F_0_) with an inter-train interval of 10 sec. We then averaged three fluorescent traces after stimulation and used peak F values to calculate ΔF/F_0_. To quantify LTE, we divided ΔF/F_0_ values obtained after associative stimulation of the AL and AFV by values obtained prior to associative stimulation.

#### Electrical stimulation

For coincident or “associative” stimulation, the AL and AFV were simultaneously stimulated with 12 trains (see above) with an inter-train interval of 5 sec, and the average peak value of the first three responses was used as the relative Ca^2+^ response during stimulation.

#### cAMP imaging

For cAMP imaging, dissected brains were incubated in HL3 medium containing 100 μM IBMX for 10 min at room temperature. IBMX and forskolin were prepared as 250 and 100 mM solutions in DMSO, and diluted in HL3 to 100 μM. CFP was excited at 457 nm and detected using a 482 ± 17.5 nm band pass filter, and YFP was detected simultaneously using a 540 ± 15 nm band pass filter. FRET changes were measured as changes in the ratio of CFP to YFP (CFP/YFP) signals. Signal changes were calculated for each pixel in the region of interest (ROI) using NIS-elements software. Images were recorded at 1 frame per second for 270 sec. Averaged CFP/YFP values in the twenty sequential frames before forskolin application were used for data normalization. Maximum CFP/YFP values were calculated by averaging CGP/YFP values in the last 120 frames of each experiment.

### Behavioral tests

The procedure for measuring olfactory memory is described elsewhere [10]. Briefly, ~100 flies were exposed sequentially to two aversive odors, 3-octanol [OCT] and 4-methylcyclohexanol [MCH], for 1 min with an interval of 45 sec between each odor exposure. When the flies were exposed by the first, CS+ odor (either OCT or MCH), they were also subjected to 1.5 sec pulses of 60 V DC electric shocks every 5 sec (US). For short-duration training, flies were exposed to the CS+ and CS- odors for 5 sec each, and one shock pulse was given during CS+ exposure [9]. To test olfactory memory, flies were placed at the choice point of a T-maze where both CS+ and CS- odors were delivered and allowed to choose between the odors. After 1.5 min, flies choosing each odor were counted, and memory was calculated as a learning index, such that a 50:50 distribution (no memory) yielded a learning index of zero and a 0:100 distribution away from the CS+ yielded a learning index of 100. For conditional expression from geneswitch drivers, flies were fed 1 mM RU486 for 4 days prior to behavior experiments.

### Quantitative RT-PCR

Total RNA was isolated from fly heads using TRIZOL reagent and reverse transcribed into cDNA using ReverTra Ace (TOYOBO) and random primers (TOYOBO). Quantitative PCR was performed using SYBR Green Realtime PCR Master Mix (TOYOBO) to measure gene expression. Primer sequences were,

*dopr* forward: 5′- CATGGGCGTTTTTCTCATCT -3′;

*dopr* reverse: 5′- AGCCAGGTGAGGATCTTGAA -3′;

*d2r* forward: 5′- GCGTGTTCATCATCTGTTGG -3′;

*d2r* reverse: 5′- TCATGCAGGAGTTGATCCAG -3′;

*dopr2* forward: 5′- GCGTGTTCATCATCTGTTGG -3′;

*dopr2* reverse: 5′- TCATGCAGGAGTTGATCCAG -3′;

*rut* forward: 5′- CGGGCTCATCCGTAATGTAG -3′;

*rut* reverse: 5′- CCCATCTCAATCACCAGTCC -3′;

*dnc* forward: 5′- CGGAGAGTCGTGGGAATTTA -3′;

*dnc* reverse: 5′- TTGCTTTTCGTTTTAGGTTTTTG -3′;

*gαs* forward: 5′- ACTGGAGCAGAACGGAGAAA - 3′;

*gαs* reverse: 5′- GAGTCCTCCGAGTTCACGTC -3′;

*g*α*i* forward: 5′- CGGTGGGTTTTGTACATCCT -3′;

*g*α*i* reverse:5′- GGATTTCTGGATGTGGTGCT -3′;

*nr1* forward: 5′-TGTTGCAAGCCGTATACCAA-3′;

*nr1* reverse: 5′-ACAGGCTATCGGAGCTTTCA-3′;

*nr2* forward: 5′- GACTATCTGGTTGCCCAGGA-3′;

*nr2* reverse: 5′- ACTTTGAGTTGCGGCTGAAT-3′;

*chat* forward: 5′-ATCATCTCGCAGTGCTTCCT-3′;

*chat* reverse: 5′- AAGACGATCTGTTCCGGATG-3′;

*ac3* forward: 5′- AAATGACGCCCCAATTACAG-3′;

*ac3* reverse: 5′- GTCCATGATTGATCCCCATC-3′;

*ac13e* forward: 5′- TTGCAGTGTCCCAGTTGATG-3′;

*ac13e* reverse: 5′- AGACCAACACCAGGATGAGC-3′;

*ac76e* forward: 5′- CAGGATGAATGACGCCCTTTCGG-3′;

*ac76e* reverse: 5′- ATGGACACAACACATGCCAGCAGC-3′;

*CG42514* forward: 5′- AATGCCTGGTTCCTTCAGTG-3′;

*CG42514* reverse: 5′- TTCCCACTAGTCCCATGAGC-3′;

### Whole mount immunohistochemistry and cell counting

MB Kenyon cells were visualized by expressing *mCD8::GFP* from the *OK107* GAL4 driver in wild-type and *chico* backgrounds. Brains were dissected, fixed in PBS containing 4 % PFA, and blocked in PBT containing 4 % BLOCK ACE (DS Pharma medical). Brains were then incubated with primary antibodies (a 1:250 dilution of chick anti-GFP polyclonal antibody (Abcam)) overnight at 4 °C. Alexa Fluor488-conjugated goat anti-chick IgG (1:400; Invitrogen) was used for the secondary antibody. Images were captured using a FV500 microscope (Olympus Corp., Japan) with a 60x objective lens. We captured confocal image stacks of all MB Kenyon cells in each brain hemisphere with a 0.1 μm interval between optical slices. Stacks of each brain were then analyzed manually to determine average cell diameters in the z-axis. This tended to be about 0.275 μm for wild-type Kenyon cells, and 0.2 μm for *chico* mutants. Optical slices at these intervals were then manually counted for GFP positive cells to quantify the total number of Kenyon cells/hemisphere.

#### Statistics

We used unpaired two-tailed student’s *t*-tests to evaluate statistical significance between two data sets and employed one-way ANOVA for multiple comparisons. Tukey’s post-hoc tests were used to determine significant individual differences after one-way ANOVA. All statistics were calculated using GraphPad Prism 5.02 (GraphPad Software).

## Abbreviations

AC: adenylyl cyclase
AFV: ascending fibers of the ventral nerve cord
ALs: antennal lobes
D1R: the D1-type dopamine receptor
*dnc*: *dunce*
FRET: fluorescence resonance energy transfer
IIS: insulin/insulin-like growth factor signaling
IUGR: intrauterine growth retardation
LTE: long-term enhancement
MBs: mushroom bodies
MCH: 4-methylcyclohexanol
nAChRs: nicotinic acetylcholine receptors
NRs: NMDA receptors
OCT: 3-octanol
PDE: phosphodiesterase
PNs: projection neurons
ROI: region of interest
*rut*: *rutabaga*
TOR: target of rapamycin kinase
US: unconditioned stimuli
VNC: ventral nerve cords
